# Cross-View Gait Recognition Method Based on Multi-Teacher Joint Knowledge Distillation

**DOI:** 10.3390/s23229289

**Published:** 2023-11-20

**Authors:** Ruoyu Li, Lijun Yun, Mingxuan Zhang, Yanchen Yang, Feiyan Cheng

**Affiliations:** 1 College of Information, Yunnan Normal University, Kunming 650500, China; roylee4463@163.com (R.L.); yangyanchen97@163.com (Y.Y.); chengfy03@163.com (F.C.); 2 Engineering Research Center of Computer Vision and Intelligent Control Technology, Department of Education, Kunming 650500, China; 3 Xi’an Institute of Applied Optics, Xi’an 710000, China; mingxuan0220@hotmail.com

**Keywords:** cross-view gait recognition, multi-teacher joint knowledge distillation, resnet

## Abstract

Aiming at challenges such as the high complexity of the network model, the large number of parameters, and the slow speed of training and testing in cross-view gait recognition, this paper proposes a solution: Multi-teacher Joint Knowledge Distillation (MJKD). The algorithm employs multiple complex teacher models to train gait images from a single view, extracting inter-class relationships that are then weighted and integrated into the set of inter-class relationships. These relationships guide the training of a lightweight student model, improving its gait feature extraction capability and recognition accuracy. To validate the effectiveness of the proposed Multi-teacher Joint Knowledge Distillation (MJKD), the paper performs experiments on the CASIA_B dataset using the ResNet network as the benchmark. The experimental results show that the student model trained by Multi-teacher Joint Knowledge Distillation (MJKD) achieves 98.24% recognition accuracy while significantly reducing the number of parameters and computational cost.

## 1. Introduction

Gait recognition, an emerging technology, utilizes people’s walking characteristics to identify and authenticate their identities. Compared with other unique biometrics, like face, fingerprint, and iris, this technology, considered one of the most promising non-invasive biometric identification methods for middle and long distances [1,2], can recognize individuals’ identity information using low-resolution gait images acquired without their active cooperation. Moreover, given the swift progression of intelligent devices and the incremental enhancement of public security infrastructure, gait recognition has found extensive applications across various domains, such as smart homes, health monitoring, and public security.

Researchers have recently started utilizing deep learning algorithms to explore gait recognition [3], with a primary focus on gait images acquired from a single view [4,5]. In practical scenarios, however, gait images collected from public places exhibit variations in view due to the random nature of pedestrian movement. Hence, it becomes essential for the network model to effectively handle gait images from multiple views [6]. Gait recognition is affected by several external factors, including carrying conditions, stride length, clothing, and camera angles. Among these factors, changes in view [7] present a particularly challenging issue. Different views introduce overlapping gait features in the images. Consequently, changes in view result in decreased recognition accuracy of the gait model. To address the impact of angle changes on gait recognition accuracy, researchers have conducted studies on cross-angle gait recognition [8,9,10,11,12,13,14,15] and achieved favorable outcomes. However, there are still some limitations to consider. The presence of a larger volume of data and more complex features in cross-view gait images necessitates a network model with enhanced learning capabilities to achieve accurate recognition. Increasing the depth and width of the network structure represents the most straightforward approach to improving the model’s feature extraction capacity. However, it also introduces challenges such as a higher number of model parameters, increased complexity, and longer training and testing times.

This paper proposes a solution to address the aforementioned problems, called Multi-teacher Joint Knowledge Distillation (MJKD). The algorithm utilizes multiple complex teacher models to train gait images from a single view. The inter-class relationships obtained from these teacher models are weighted and integrated into the inter-class relationship set. This set is then used to guide the training of a lightweight student model, enhancing its gait feature extraction ability and achieving higher recognition accuracy. To verify the effectiveness of MJKD, extensive experiments are conducted on the CASIA_B dataset using the ResNet network. The CASIA_B dataset is a multi-view human gait dataset collected by the Institute of Automation of the Chinese Academy of Sciences, which has the advantages of large-scale, multi-view, and multiple walking states. It stands as one of the most extensively utilized public gait datasets in contemporary research. ResNet, a deep neural network architecture, has achieved notable success in the realm of gait recognition technology owing to its outstanding performance and superior characteristics. The deep structure of ResNet enables it to extract features from complex gait patterns to improve recognition performance. The incorporation of residual connections within ResNet serves as a strategic measure to effectively mitigate the vanishing gradient problem inherent in the training process of deep networks. This enhancement contributes to heightened stability and efficiency during the training phase. The experimental results confirm that the student model trained with MJKD achieves higher recognition accuracy while significantly reducing the number of parameters and calculation costs.

The remainder of this paper is organized as follows: Section 2 introduces the related research on gait recognition and knowledge distillation. Section 3 provides a detailed explanation of the proposed MJKD method. In Section 4, experiments are conducted using the CASIA_B gait dataset [16] to validate the effectiveness of the proposed method. Finally, Section 5 concludes the paper by summarizing the key findings and contributions.

## 2. Related Work

This paper primarily focuses on two aspects: gait recognition research and knowledge distillation research. We will examine both of these areas in detail, exploring them from different perspectives.

### 2.1. Gait Recognition

Currently, gait recognition methods can be broadly categorized into two types: appearance-based and model-based [17]. The model-based approach utilizes the estimated human body structure as its output. Jinkai Zheng et al. [18] proposed the use of three-dimensional geometric information from the SMPL model to enhance gait feature learning. In a similar vein, Torben Teepe et al. [19] employed human pose estimation to directly estimate bone pose from RGB images and introduced graph convolution for gait representation learning based on 2D bones. While model-based methods exhibit robustness to noise factors such as carrying conditions and clothing, accurately modeling the gait remains a challenging task, and these methods do not perform well at low resolutions. With the emergence of deep learning, appearance-based gait recognition methods have gained increasing attention. These methods directly learn shape features from the input video, making them suitable for low-resolution conditions. Additionally, based on the type of input data [20], proposed approaches can be categorized into template-based and sequence-based methods. In template-based methods, gait features are extracted from a single gait image, such as the gait energy image (GEI) [21] or other GEI-like template images [22,23]. On the other hand, the sequence-based approach builds a model from the gait contour sequence and utilizes temporal modeling to encode information across time. Wu et al. introduced three ConvNets with diverse structures and conducted a series of experiments, effectively enhancing cross-view angle gait recognition. Moreover, generative models, including autoencoders [24] and generative adversarial networks [25,26,27], have also been employed in gait recognition studies.

### 2.2. Knowledge Distillation

Knowledge distillation [28] is an algorithm based on the student-teacher learning paradigm. Currently, knowledge distillation can be categorized into three types: model output as knowledge, network learning features as knowledge, and network feature relationships as knowledge. In 2014, Ba et al. [29] proposed a method that utilizes a teacher model to supervise the learning of a student model. The student model is trained using logits before the softmax layer of the teacher model. The optimization of the loss between the teacher model’s logits output and the student model’s logits results in improved recognition accuracy for the student model. One of the most influential works in knowledge distillation is by Hinton et al. [30]. They trained students by minimizing the cross-entropy loss between the softmax layer output of the teacher model and the student model, as well as minimizing the cross-entropy between the predicted values of the student model and the actual labels. This approach ensures that the student model not only fits the ground truth of the training data but also aligns with the probability distribution of the teacher model’s output. In another study, Sau et al. [31] proposed adding Gaussian noise with an average value of 0 and a standard deviation of σ to the logits of the teacher model, simulating a multi-teacher scenario. The aforementioned literature belongs to the knowledge distillation method, which considers model output as knowledge. Romero et al. [32] proposed a knowledge distillation method based on the features of network learning. They achieved this by extracting the output of the middle layer of both the teacher and student models and aiming to make them as close as possible. To map the hidden layer of the student model to the prediction of the hidden layer of the teacher model, additional parameters were introduced. Building upon the FitNets approach, Shan You et al. [33] further improved the knowledge distillation technique. They employed a triplet loss to impose constraints on the middle layer output of multiple teacher models. Additionally, they utilized a voting strategy to unify the dissimilar information from these multiple teacher models. In their study, Junho Yim et al. [34] proposed another knowledge distillation method based on the network feature relation. They initially adjusted the parameters of the student model according to the flow of solution procedure (FSP) matrix of the teacher model. This adjustment aimed to make the interlayer relationship of the student model similar to that of the teacher model. Subsequently, the parameters of the student model were fine-tuned using the original loss function.

## 3. Multi-Teacher Joint Knowledge Distillation

The knowledge distillation (KD) method typically comprises both a student model and a teacher model. The teacher model utilizes inter-class relationships acquired during training to direct the training of the student model. Yet, within the domain of cross-view gait recognition, the cross-view gait image integrates gait images from distinct single views, housing a larger volume of data and a more comprehensive array of features. The gait features within images from various views exhibit overlaps, thereby yielding variations in gait characteristics. Employing the KD method results in a diminished richness of gait features extracted by an individual teacher model, and the inter-class relationships established during training fail to effectively guide the training of the student model. To enhance the feature extraction ability of lightweight models in cross-view gait recognition, we propose a method called Multi-teacher Joint Knowledge Distillation (MJKD). This approach is based on the “student-teacher learning paradigm”. We employ multiple complex teacher models to train gait images captured from a single view. We assign weights to the inter-class relationships obtained from this training and integrate them into a unified set of inter-class relationships. Subsequently, we utilize this set to guide the training of the lightweight student model. The goal is to improve the student model’s ability to extract gait features and achieve higher recognition accuracy.

**Figure 1:**
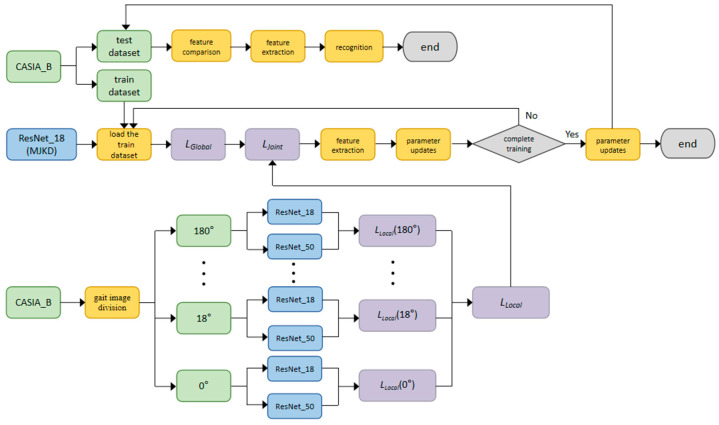
The flowchart of the Multi-teacher Joint Knowledge Distillation framework. (Green is the dataset module, blue is the network model module, purple is the loss function module, yellow is the module of data division, data loading, feature extraction, feature comparison, parameter updating and recognition, grey is the operation select and end module).

The “student-teacher learning paradigm” serves as a fundamental framework for transferring inter-class relationships. In essence, the quality of the student model’s ability to extract inter-class relationships obtained by the teacher model during training hinges on the teacher-student structure. The richer the inter-class relationships acquired by the teacher model, the stronger the student model’s feature extraction capability after guided training. In terms of the human learning process, students undergo comprehensive exams covering various subjects. To address the challenges across different subjects more effectively, they require teachers with expertise in specific subjects. By acquiring knowledge across diverse subjects, students enhance their proficiency in dealing with a variety of subjects. Motivated by this analogy, cross-view gait recognition is treated as training the student model with gait images captured from multiple views. To facilitate the student model in extracting pertinent gait features from images taken from different views, it is necessary to employ multiple teacher models to individually train gait images from a single view. The resulting inter-class relationships are weighted and integrated into a unified set, which is then used to guide the training of lightweight student models. The flowchart of the algorithm is shown in Figure 1.

### 3.1. Multi-Teacher Joint Knowledge Distillation Framework

**Figure 2:**
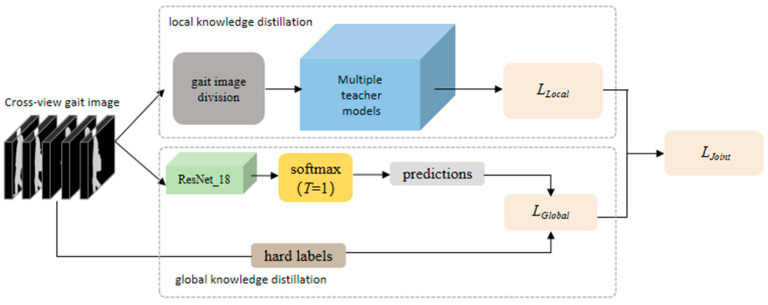
Multi-teacher Joint Knowledge Distillation framework. (Green is the student model module, blue is the multi-teacher model module, dark gray is the data division module, brown is the hard label module of the data, light gray is the student model prediction module, yellow is the softmax output module, pink is the loss function module).

**Figure 3:**
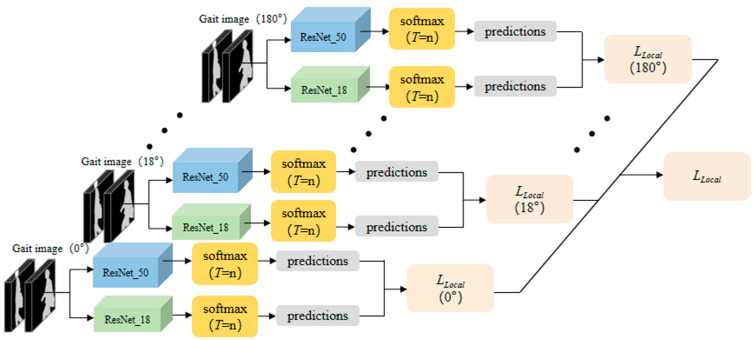
Local knowledge distillation for single-view gait features. (Green is the student model module, blue is the teacher model module, yellow is the softmax output module, gray is the prediction module, and pink is the loss function module).

As shown in Figure 2 and Figure 3, ResNet is utilized as the underlying network [35], with ResNet_50 serving as the teacher model and ResNet_18 as the student model. The parameter count of ResNet_50 is 22.66 M, whereas ResNet_18 has 10.72 MB parameters. This paper introduces MJKD, which comprises three components: local knowledge distillation for single-view gait features, global knowledge distillation for cross-view gait features, and joint knowledge distillation. In local knowledge distillation, the loss is calculated between the output of multiple teacher models and the student models for each single viewing angle gait image. This step aims to distill the specific knowledge from the teachers to the student regarding gait recognition. On the other hand, global knowledge distillation computes the loss between the output of the student model and the ground truth of the cross-view gait image. This process helps the student model learn global features from the ground truth and refine its understanding of cross-view gait characteristics. To combine these two types of knowledge distillation, we introduce combined distillation, which measures the difference between the losses obtained from local and global knowledge distillation. By fusing the losses, the training process effectively transfers knowledge from multiple teacher models to the student model. Consequently, the student model is trained more efficiently, leading to an improvement in the recognition accuracy.

MJKD leverages the softening output of the softmax layer as knowledge to guide the training of the student model. The softmax function facilitates the compression of a K-dimensional vector, denoted as z, into another K-dimensional real vector σ (z). This transformation ensures that each element of the vector ranges between (0, 1), with the sum of all elements equaling 1. By softening the input data through the softmax layer, the probabilities associated with each class can be assigned to the output results. This approach allows for a more precise description of the relationship between classes obtained by the teacher model. The softmax function is shown in Formula (1).

(1) $$\begin{array}{c} q_{i}=\frac{\exp\left(z_{i}\right)}{\sum_{j}exp\left(z_{j}\right)} \end{array}$$

In the given formula, we have $q_{i}$ representing the probability of softmax output in category i, $z_{i}$ representing the logits output in category i, and $z_{j}$ representing the logits of a total of j categories. The softmax layer’s softened output reveals that certain negative labels are more likely to correspond to negative labels compared with the real labels, which are neither 0 nor 1. This observation forms the basis for the training method of MJKD, where each sample contributes more information to the student model than in traditional training methods. However, there is an issue when the output probability distribution entropy of softmax is small. In such cases, the value of each negative label approaches 0, implying that their contribution to the loss function is close to 0 as well. To address this, a distillation temperature T is introduced into the MJKD process to controllably expand or contract the probability distribution associated with positive and negative labels. This concept is represented by Formula (2).

(2) $$\begin{array}{c} q_{i}=\frac{\exp\left(\frac{z_{i}}{T}\right)}{\sum_{j}exp\left(\frac{z_{j}}{T}\right)} \end{array}$$

By comparing Formulas (1) and (2), we observe that Formula (2) is equivalent to Formula (1) when the value of T is 1. When the T value increases, the probability distribution of the softmax layer output becomes smoother, resulting in a larger entropy for the distribution. As a result, the positive labels of the correct category are relatively reduced, while the negative labels of the incorrect categories are relatively amplified. This amplification of negative labels carries more learnable information and leads to the model training paying extra attention to the negative labels. On the other hand, when the T value decreases, the difference in the probability distribution output of the softmax layer becomes larger. A smaller entropy for the distribution implies that the information carried by the negative labels of the incorrect category is relatively compressed. Consequently, the model training predominantly focuses on the positive labels. The effect of different distillation temperatures T on the performance of MJKD is further discussed in Section 4.7.3.

### 3.2. Local Knowledge Distillation for Single-View Gait Features

In order to effectively extract the gait features of each single viewing angle, MJKD uses multiple teacher models and student models to train the images of each single viewing angle respectively. Through continuous iterative training, the probability distribution of the output of the teacher model at the distillation temperature T is as similar as possible to that of the student model at the distillation temperature. As shown in Figure 3, in the local knowledge distillation, the cross-view gait image is divided into 11 single-view gait data points according to the angle. Each set of single-view gait data is trained by a teacher model and a student model, and the predicted results obtained from the training are performed by loss calculation. Finally, the weights of the 11 local losses are averaged and summed to obtain $L_{Local}$. Because there is no overlap of features in the same view angle, the gait features extracted from the same view angle are more abundant. Local loss $L_{Local}$ is shown in Formula (3).

(3) $$L_{Local}=-\sum_{i}^{N}p_{i}^{T}\log(q_{i}^{T})=-\sum_{i}^{N}\frac{\exp\left(\frac{v_{i}}{T}\right)}{\sum_{k}exp\left(\frac{v_{k}}{T}\right)}\log\left(\frac{\exp\left(\frac{z_{i}}{T}\right)}{\sum_{k}exp\left(\frac{z_{k}}{T}\right)}\right)$$

In the formula, several variables have specific roles.N represents the total number of labels, while $v_{i}$ and $z_{i}$ correspond to the logits output by the teacher model and the student model, respectively, for category i. Furthermore, $v_{k}$ and $z_{k}$ represent the logits output by the teacher model and the student model, respectively, for the entire set of k categories. Additionally, $p_{i}^{T}$ and $q_{i}^{T}$ denote the probability output of the teacher model and the student model’s softmax layer for category i when the distillation temperature T is applied. The local loss refers to the probability distribution that minimizes the output difference between the student model and the teacher model. During training, the soft target generated by the teacher model is utilized. The student model is guided by this soft target, and through continuous iterative training, the disparity between the student model and the soft target gradually reduces.

### 3.3. Global Knowledge Distillation for Gait Features across Viewing Angles

Global knowledge distillation is distinct from local knowledge distillation as it focuses on extracting gait features from cross-view angle gait images, enabling the establishment of global features for cross-view angle gait recognition. In this approach, the probability distribution of the softmax layer output from the student model is compared with the ground truth of the cross-view gait data, generating a loss. Through continuous iterative training, the discrepancy between the softmax output and the ground-truth is gradually minimized. This loss is represented as the global loss $L_{Global}$, as shown in Formula (4).

(4) $$\begin{array}{c} L_{Global}=-\sum_{i}^{N}c_{i}\log\left(q_{i}^{1}\right)=-\sum_{i}^{N}c_{i}\log\left(\frac{\exp\left(z_{i}\right)}{\sum_{k}exp\left(z_{k}\right)}\right) \end{array}$$

In the formula above, $c_{i}$ represents the ground truth value for category i. $c_{i}$ ∈ {0,1}, where a positive label is denoted as 1 and a negative label as 0. $q_{i}^{1}$ represents the logits output by the student model for category i., with a distillation temperature of T = 1. The purpose of the global loss $L_{Global}$ is to ensure that the probability distribution generated by the student model consistently aligns with the true distribution of ground truth when training on cross-view gait images.

### 3.4. Joint Knowledge Distillation

Although gait images with a single view angle allow for the extraction of more abundant gait features, there is a lack of global recognition for cross-view gait analysis. While global loss $L_{Global}$ plays a significant role in cross-view gait recognition, it is not suitable for extracting gait features due to the overlap of features across different viewing angles during training. In the context of human learning, students possess the capability to handle various subject-specific problems, but they often struggle with comprehensive problem-solving. To excel in comprehensive examinations, students need both subject-specific proficiency and the ability to tackle comprehensive questions. Hence, in joint knowledge distillation, the joint loss $L_{joint}$ is calculated as the weighted sum of the local loss $L_{Local}$ and the global loss $L_{Global}$, as outlined in Formula (5).

(5) $$L_{joint}=\frac{1-\alpha }{n}\sum_{i=1}^{n}L_{Local(i)}+\alpha L_{Global}$$

The above formula introduces the variables used in the equation. In this case, α represents the weight value assigned to the two losses, with α ∈ (0, 1). n refers to the number of local losses involved. The joint loss $L_{Joint}$ is obtained by taking the weighted sum of each local loss $L_{Local}$ and the global loss $L_{Global}$, after assigning the appropriate weights on average. The impact of different weights α on the performance of MJKD is discussed in detail in Section 4.7.2.

## 4. Experiment and Analysis

### 4.1. Experimental Data

**Figure 4:**
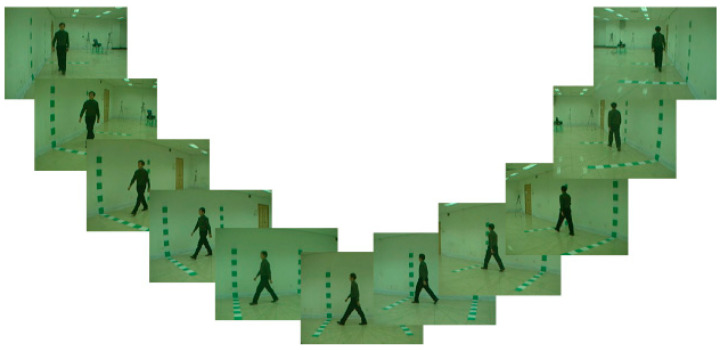
CASIA_B multi-view gait dataset.

To advance the research and development of gait recognition technology, the Institute of Automation at the Chinese Academy of Sciences has established the CASIA gait database, which provides a standard benchmark for researchers to evaluate, improve, and compare gait recognition algorithms. The database encompasses three datasets: CASIA_A (a small-scale human gait dataset), CASIA_B (a multi-view human gait dataset), and CASIA_C (a single-view infrared gait dataset). This paper utilizes CASIA_B, a multi-view human gait dataset within the CASIA Gait Database of the Institute of Automation of the Chinese Academy of Sciences. It was collected in January 2005 and stands as one of the most widely used public gait datasets. The CASIA_B dataset offers several advantages, including its large-scale nature, multi-view, and multiple walking states. It consists of 124 subjects, each with 11 views from various angles (0°, 18°, 36°, … 180°). Each view includes three walking conditions: normal walking state (NM), walking with coat (CL), and walking with bag (BG). Figure 4 presents the schematic diagram of subject number 1 captured at 11 different viewing angles while walking in the normal walking state (NM).

### 4.2. Experimental Design

Cross-view gait recognition poses challenges, as the gait features present in images captured from different viewing angles often overlap. Consequently, network models struggle to effectively extract these features, resulting in unsatisfactory gait recognition accuracy. This experiment is divided into four parts. The first part evaluates the effectiveness of MJKD and analyzes the obtained results. In the second part, we compare the recognition accuracy of ResNet_18 trained by MJKD with other more advanced gait recognition models. The third part involves training and testing the ResNet network using both MJKD and standard knowledge distillation techniques. We thoroughly compare and analyze the results, along with the training and testing process and duration. Part 4 focuses on conducting the ablation experiment. For this experiment, the training and validation sets are divided in a 7:3 ratio. The gait images in MJKD are categorized into 11 viewing angles based on the shooting view. Each view’s gait image is inputted into the model according to algorithm requirements. Furthermore, in this experiment, the gait images captured at the 11 viewing angles are randomly combined to form cross-view gait images. This approach ensures that the model’s effectiveness aligns more closely with real-life scenarios. The configuration of the environment for this experiment is presented in Table 1.

### 4.3. Effectiveness Analysis of Multi-Teacher Joint Knowledge Distillation (MJKD)

As shown in Table 2, when compared with the directly trained teacher model ResNet_50, the ResNet_18 model trained using MJKD exhibited significant reductions in Params and FLOPs, by 2.11 times and 2.27 times, respectively. Moreover, the model achieved a slight improvement in recognition accuracy of 0.04%. In this analysis, it can be seen that although the teacher model ResNet_50 achieves a recognition accuracy of 98.21%, the model complexity is higher and the number of parameters is larger. In comparison with the directly trained student model ResNet_18, the ResNet_18 model trained using MJKD exhibited a noteworthy improvement of 9.76% in recognition accuracy while maintaining the same Params and FLOPs. However, the analysis reveals that despite ResNet_18 having lower Params and FLOPs, its inherent learning ability limits its recognition accuracy to only 88.48% in the cross-view gait recognition task. Therefore, experiments show that MJKD can help student model ResNet_18 extract gait features more effectively, guided by teacher model ResNet_50, thereby improving their own recognition accuracy.

### 4.4. Comparison with Recent Technologies

Table 3 presents a comparison of the recognition accuracy between the proposed ResNet_18 model trained using MJKD and several advanced gait recognition models (SPAE, GaitGANv1, GaitGANv2, Deep CNN, J-CNN, GaitSit, GaitNet, and GaitPart). Analyzing the results in Table 3 reveals that the ResNet_18 model trained with MJKD outperforms the other models, exhibiting the highest recognition accuracy. This superiority can be attributed to the unique characteristics of MJKD. This algorithm incorporates both interclass relationships derived from multiple teacher models operating on gait images of individual views and global information obtained from student models during the training process, specifically for cross-view gait images. As a result, ResNet_18 utilizing MJKD training exhibits enhanced gait feature extraction abilities, thereby achieving superior recognition accuracy in cross-view gait recognition tasks.

### 4.5. Comparative Analysis of Recognition Accuracy of Different Knowledge Distillation Methods

**Figure 5:**
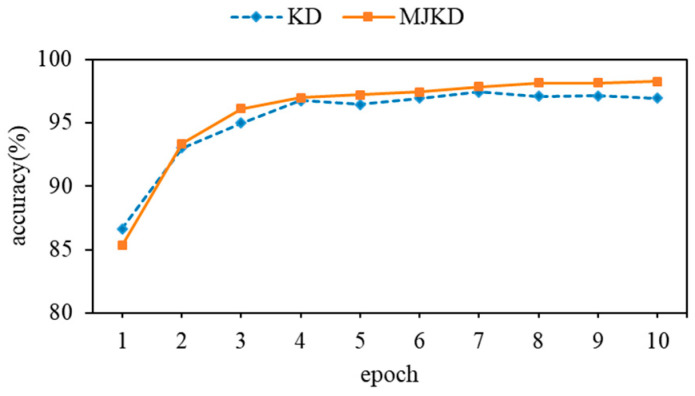
Comparison of results on the ResNet network.

To evaluate the effectiveness of the proposed MJKD approach, we conducted training and testing experiments using both Multi-teacher Joint Knowledge Distillation (MJKD) and knowledge distillation (KD) techniques on the ResNet network. The results shown in Table 4 indicate that ResNet_18 trained using KD achieves a recognition accuracy of 96.95%, while ResNet_18 trained using MJKD achieves a notably higher recognition accuracy of 98.24%. This is a significant improvement of 1.59%. Furthermore, Figure 5 illustrates that ResNet_18 trained with MJKD exhibits faster convergence and less fluctuation in recognition accuracy during the same training iterations.

**Figure 6:**
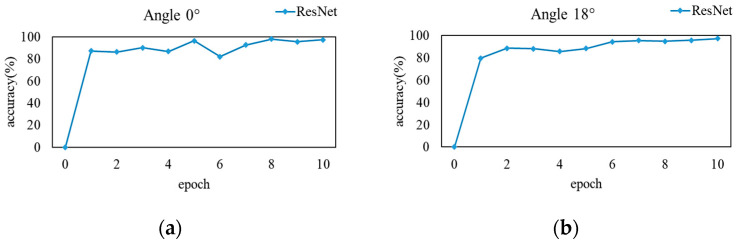

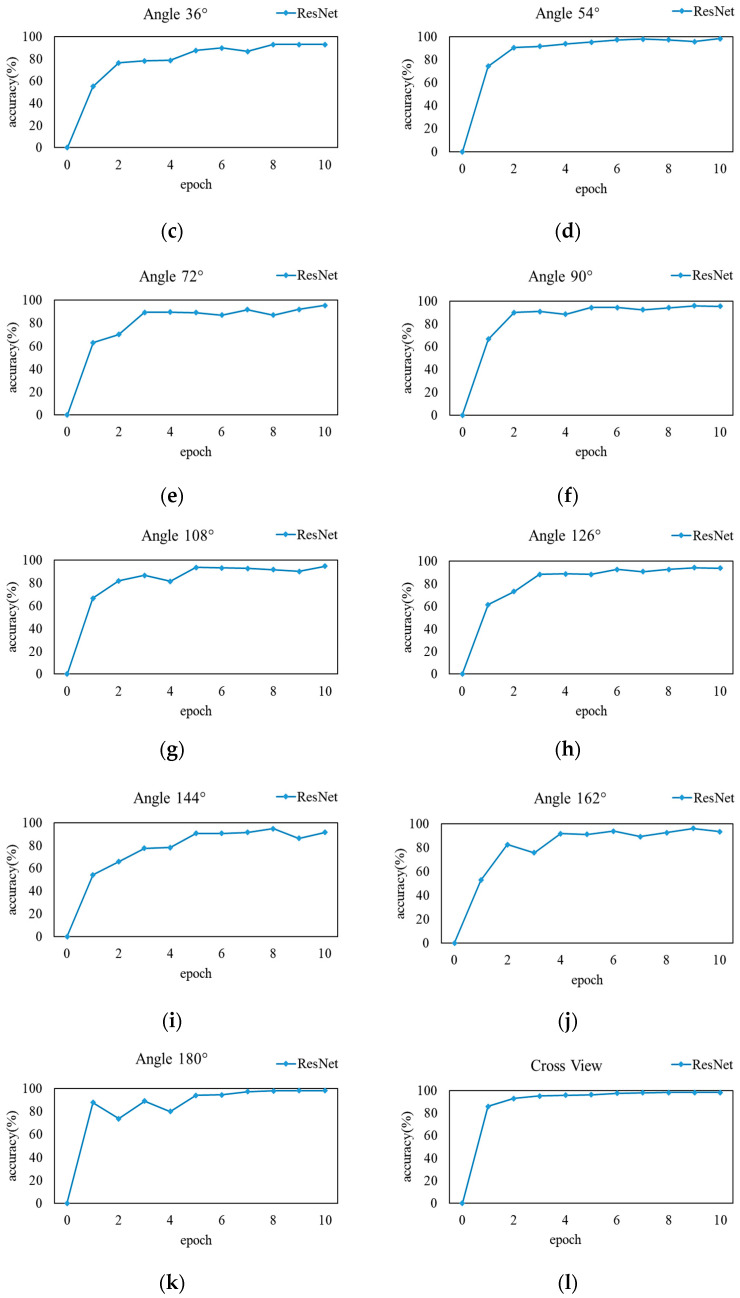
Training results of the network model under different views. ((**a**–**k**) are line charts of the recognition accuracy of a single teacher module after training on the 0° to 180° view datasets, respectively. (**l**) is line chart of the recognition accuracy of a single teacher module after training on the cross-view dataset).

In the above experiments, both knowledge distillation (KD) and Multi-teacher Joint Knowledge Distillation (MJKD) techniques were employed, utilizing the softened output of the softmax layer as the guiding knowledge for training the student models. However, there exists a distinction in the gait features extracted by the softmax layer’s softening output between KD and MJKD. The softening output of KD encompasses knowledge derived from the inter-class relationships attained through teacher model training with cross-view gait images. On the other hand, the softened output of MJKD comprises the aggregation of inter-class relationships obtained by training 11 teacher models using 11 single-view gait images. As human posture varies at different view angles, gait features tend to overlap. Therefore, compared with gait features spanning multiple views, the teacher model extracts a greater number of gait features from each individual view, ultimately enhancing the student model’s ability to extract features, guided by this training. The training process of the teacher model, encompassing both single-view and cross-view gait images, is depicted in Figure 6.

### 4.6. Comparative Analysis of Training and Test Duration of Different Knowledge Distillation Methods

**Figure 7:**
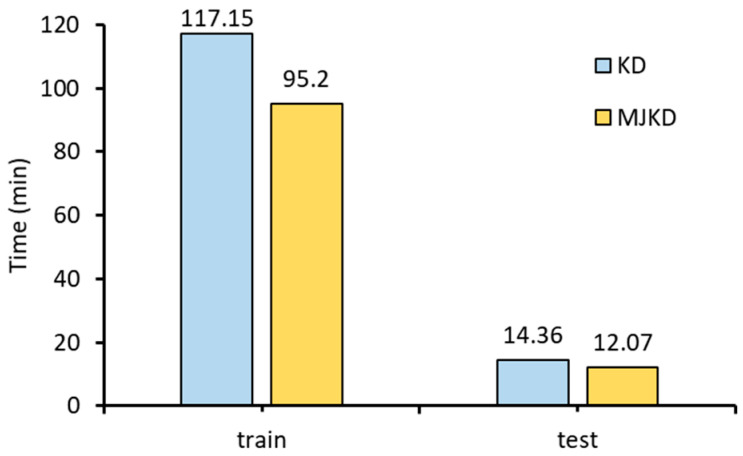
Comparison of network model training and testing time.

Training and test duration are important indicators for measuring the performance of a model. In this study, we aim to evaluate the performance of ResNet trained using MJKD. Both MJKD and KD methods were employed to train and verify ResNet, and the results are presented in Figure 7. The analysis reveals that the training time for ResNet_18 trained with KD is 117.15 min, whereas with MJKD it is reduced to 95.2 min, resulting in a 1.23 times reduction in duration. The ResNet_18 trained with KD took 14.36 min, while the one trained using MJKD had a test duration of 12.07 min, which is 1.19 times shorter. From the above data, it is evident that ResNet_18 trained using MJKD exhibits faster reasoning speed and reduced time costs during both the training and testing phases.

### 4.7. Ablation Experiment

#### 4.7.1. Analysis of the Influence of Each Knowledge Distillation Module on the Recognition Accuracy

To assess the effectiveness of the proposed MJKD approach, we conducted tests on both local and global knowledge distillation within MJKD. These tests involved using local loss $L_{Local}$ and global loss $L_{Global}$ as the loss functions to train ResNet_18. The resulting recognition accuracy rates can be found in Table 5. The recognition accuracy of ResNet_18 when using only local loss $L_{Local}$ is a mere 0.69%. This low accuracy highlights the limitations of local knowledge distillation, which incorporates inter-class relationships obtained from multiple teachers trained on gait images from each single view. It is important to note that due to the lack of ground truth constraints, these inter-class relationships might deviate from the actual data distribution. Furthermore, the introduction of the distillation temperature T into the softmax layer of local knowledge distillation amplifies the probability distribution of negative labels. While this increase in learnable information can be beneficial, it also heightens the risk of deviating from the true data distribution. The recognition accuracy of ResNet_18 significantly improves to 94.34% when using only global loss $L_{Global}$. From this analysis, we can see that using only global loss $L_{Global}$ is equivalent to adding pre-training weight to ResNet_18, so the recognition accuracy is improved. Moreover, when considering different loss approaches, the recognition accuracy of ResNet_18 reaches its pinnacle with the utilization of joint losses $L_{joint}$. Notably, this outperforms the use of either only local losses $L_{Local}$ or only global losses $L_{Global}$. Consequently, the experimental results validate MJKD employing joint loss $L_{joint}$ as the optimal approach.

#### 4.7.2. Analysis of the Influence of Weight on Recognition Accuracy

**Figure 8:**
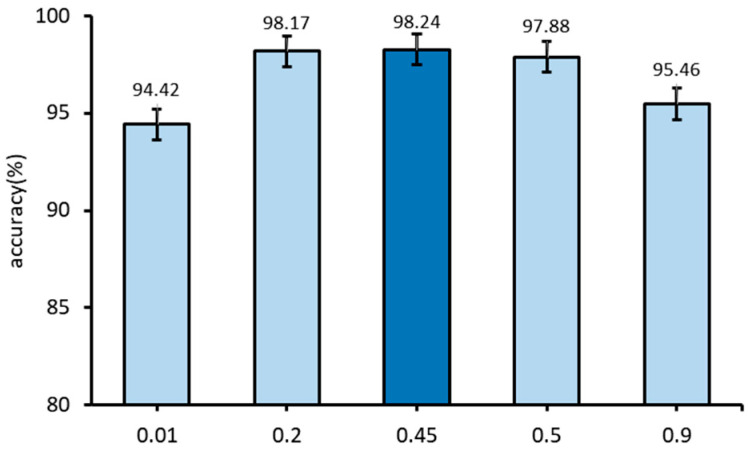
Comparison of recognition accuracy of different weight parameters. (The light blue represents the recognition accuracy when the weight α is 0.01, 0.2, 0.5 and 0.9 respectively, dark blue represents the recognition accuracy when the weight α is 0.45).

α is the weight parameter of joint loss $L_{joint}$. The weight parameter α plays a critical role in determining the balance between local loss $L_{Local}$ and global loss $L_{Global}$ in model training. When the α is set to 0.5, the contribution of local loss $L_{Local}$ and global loss $L_{Global}$ is equal. If the α of global loss $L_{Global}$ is less than 0.5, it becomes larger, indicating that the model training emphasizes the information provided by the global loss $L_{Global}$. Conversely, when the α exceeds 0.5, the weight of local loss becomes larger, indicating a higher emphasis on the information provided by the local loss $L_{Local}$. Figure 8 shows the results of recognition accuracy under different weight parameters α. It can be seen that, with the increase of the value of α, the recognition accuracy increases first and then decreases. In the case of maximum or minimum, the recognition accuracy changes greatly, and the changes in other cases have little impact on the recognition accuracy. In this experiment, when the weight parameter α is 0.45, the recognition accuracy is the highest.

#### 4.7.3. Analysis of the Influence of Distillation Temperature on Recognition Accuracy

**Figure 9:**
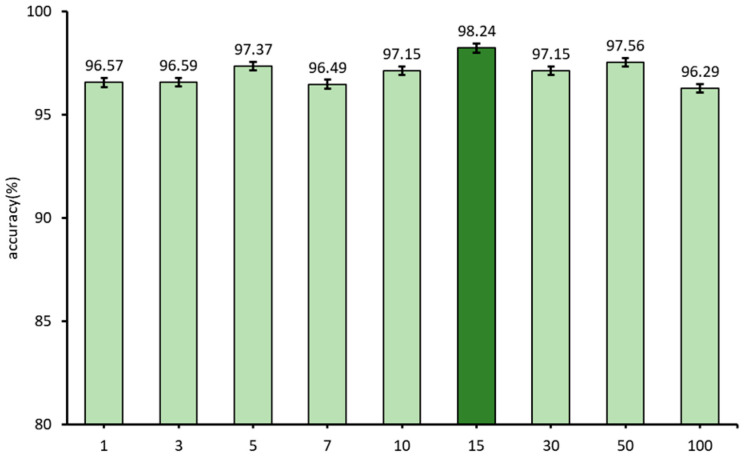
Comparison of recognition accuracy of different distillation temperatures T. (Light green represents the recognition accuracy when the distillation temperature T is 1, 3, 5, 7, 10, 30, 50 and 100 respectively, dark green represents the recognition accuracy when the distillation temperature T is 15).

The MJKD algorithm leverages the softening output of the softmax layer to guide student model training. The distillation temperature T is a crucial factor that can either amplify or diminish the positive and negative labels in the softening output. As the value of T becomes larger, the probability distribution of the output of the softmax layer tends to be smoother, the entropy of the distribution is larger, the positive labels of the correct category are relatively reduced, the negative labels of the incorrect categories are relatively amplified, the learnable information carried will be relatively amplified, and the model training will pay extra attention to the negative labels. When the value of T becomes smaller, the difference in probability distribution output by the softmax layer becomes larger, the entropy of its distribution is smaller, the information carried by the negative label of the incorrect class will be relatively compressed, and the model training will mainly focus on the positive label. Figure 9 shows the recognition accuracy results at different distillation temperatures, and it can be seen that the distillation temperature has little influence on the recognition accuracy. In this experiment, the best identification accuracy was achieved when the distillation temperature T was 15.

#### 4.7.4. Joint Analysis of the Influence of Distillation Temperature and Weight on Recognition Accuracy

**Figure 10:**
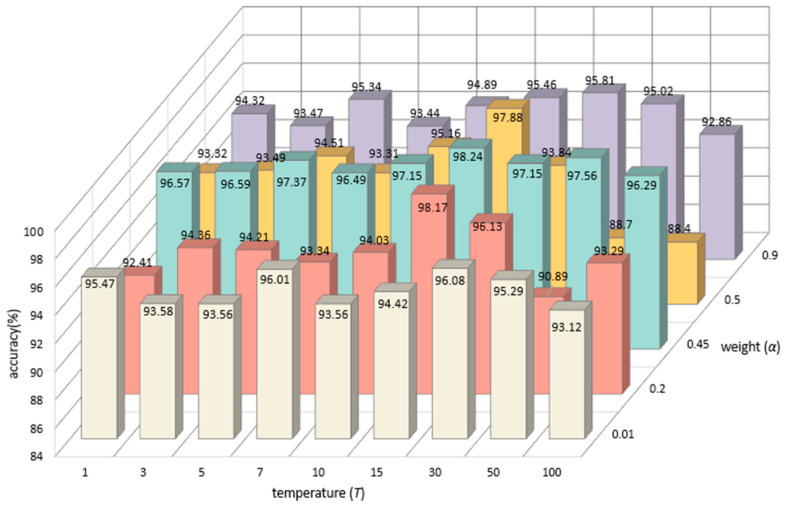
Joint comparison of recognition accuracy for different distillation temperatures T and weights α. (White represents the recognition accuracy at different distillation temperatures T when the weight α is 0.01. Red represents the recognition accuracy at different distillation temperatures T when the weight α is 0.2. Green represents the recognition accuracy at different distillation temperatures T when the weight α is 0.45. Yellow represents the recognition accuracy at different distillation temperatures T when the weight α is 0.5. Purple represents the recognition accuracy at different distillation temperatures T when the weight α is 0.9).

Building upon the insights gleaned from Section 4.7.2 and Section 4.7.3, a comprehensive joint analysis is undertaken to assess the combined influence of variables T and α on recognition accuracy. In Section 4.7.2, notable fluctuations in recognition accuracy are observed when α attains either extreme, with optimal results achieved when weight parameter α is 0.45. In Section 4.7.2, the recognition accuracy results at different distillation temperatures T are shown, and it can be seen that the distillation temperature T has little influence on the recognition accuracy. In this experiment, the best identification accuracy was achieved when the distillation temperature T was 15. Figure 10 shows the recognition accuracy results for different temperatures T and different weights α. The recognition accuracy is low when the weight α and temperature T are extremely high or extremely low, and the best results are achieved when α is 0.45 and the distillation temperature T is 15. The empirical findings in this section exhibit concordance with those elucidated in Section 4.7.2 and Section 4.7.3. Additionally, the experiments undertaken in this specific section serve to reinforce and substantiate the rationale behind the experiments delineated in Section 4.7.2 and Section 4.7.3. This iterative validation contributes to the robustness and reliability of the experimental methodology employed across the entire investigation.

#### 4.7.5. Analysis of the Influence of Learning Rate on Recognition Accuracy

**Figure 11:**
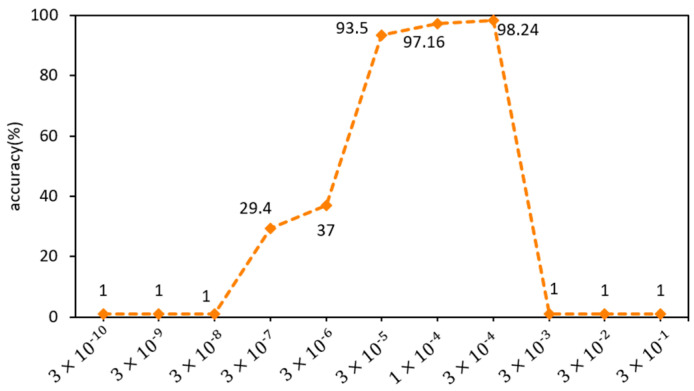
Comparison of recognition accuracy of different learning rates.

The influence of different learning rates on recognition accuracy is illustrated in Figure 11. The graph indicates that, initially, as the learning rate increases, the recognition accuracy improves. However, beyond a certain point, a further increase in the learning rate leads to a decline in accuracy. When the learning rate is too small, the model fitting speed becomes slow, resulting in low recognition accuracy after 10 epochs of iterative training. On the other hand, when the learning rate is too large, the model fails to converge. In this experiment, the highest recognition accuracy was achieved when the learning rate was set to 3 × 10^−4^.

## 5. Conclusions

Aiming at challenges such as the high complexity of the network model, numerous parameters, and the slow speed of training and testing in cross-view gait recognition, this paper proposes a solution: MJKD. The algorithm employs multiple complex teacher models to train gait images from a single view, extracting inter-class relationships that are then weighted and integrated into the set of inter-class relationships. These relationships guide the training of a lightweight student model, improving its gait feature extraction capability and recognition accuracy. To validate the effectiveness of the proposed MJKD, the paper performs experiments on the CASIA_B dataset using the ResNet network as the benchmark. The experimental results demonstrate that the lightweight student network, trained through MJKD, achieves higher recognition accuracy in cross-view angle gait recognition tasks. Additionally, the model parameters are reduced by 2.11 times, the model complexity is reduced by 2.27 times, and the training and verification times are reduced by 1.23 and 1.19 times, respectively. As a result, the MJKD algorithm not only ensures a smaller number of parameters and lower model complexity but also improves operational speed and achieves better recognition accuracy in cross-view gait recognition tasks. In future work, we plan to explore a knowledge distillation method that utilizes the features from the middle layer of the network as knowledge. This method will be combined with the MJKD presented in this paper. This approach aims to further investigate cross-view gait recognition tasks and enhance the application ability of gait recognition in real-world scenarios.

## Figures and Tables

**Table 1:** Experimental environment configuration.

Environment Name	Configure Parameters
operating system	Windows 10
CPU	Intel i7-10700F
CPU Frequency	2.90 GHz
memory	48 GB
GPU	NVIDIA RTX 3060
GPU memory	12 GB
Graphics card frequency	1320–1777 MHz
IDE Environment	PyCharm Community
Compiled language	Python 3.7
Open source framework	PyTorch 1.7

**Table 2:** Experimental results of ResNet on cross-view angle gait images.

Network	Method	ACC (%)	FLOPs (G)	Params (M)
ResNet	ResNet_50 (teacher)	98.21	1.34	22.66
	ResNet_18 (student)	88.48	0.59	10.72
	ResNet_18 (MJKD)	98.24	0.59	10.72

**Table 3:** Comparison of recognition accuracy with different technologies.

Method	ACC (%)
SPAE [8]	71.39
GaitGANv1 [9]	70.95
GaitGANv2 [10]	72.42
Deep CNN [11]	90.75
J-CNN [12]	73.57
GaitSet [13]	84.19
GaitNet [14]	77.54
GaitPart [15]	89.13
ResNet_18 (MJKD)	98.24

**Table 4:** Comparison of recognition accuracy rates using different knowledge distillation methods.

Network	Method	ACC (%)
ResNet	ResNet_18 (KD)	96.95
	ResNet_18 (MJKD)	98.24

**Table 5:** Comparison results of recognition accuracy of each knowledge distillation module.

Network	Method	ACC (%)
ResNet_18	$L_{Local}$	0.69
	$L_{Global}$	94.34
	$L_{Local}+L_{Global}$	98.24

## Data Availability

The dataset used in the experiment described in this article comes from the CASIA gait database provided by the Institute of Automation of the Chinese Academy of Sciences. The dataset can be found in the CASIA gait database of the Chinese Academy of Sciences: http://www.cbsr.ia.ac.cn/china/Gait%20Databases%20CH.asp.
